# Sequencing and comparative analysis of fugu protocadherin clusters reveal diversity of protocadherin genes among teleosts

**DOI:** 10.1186/1471-2148-7-49

**Published:** 2007-03-30

**Authors:** Wei-Ping Yu, Kenneth Yew, Vikneswari Rajasegaran, Byrappa Venkatesh

**Affiliations:** 1 Gene Regulation Laboratory, National Neuroscience Institute, 11 Jalan Tan Tock Seng 308433, Singapore; 2 Institute of Molecular and Cell Biology, 61 Biopolis Drive 138673, Singapore

## Abstract

**Background:**

The synaptic cell adhesion molecules, protocadherins, are a vertebrate innovation that accompanied the emergence of the neural tube and the elaborate central nervous system. In mammals, the protocadherins are encoded by three closely-linked clusters (α, β and γ) of tandem genes and are hypothesized to provide a molecular code for specifying the remarkably-diverse neural connections in the central nervous system. Like mammals, the coelacanth, a lobe-finned fish, contains a single protocadherin locus, also arranged into α, β and γ clusters. Zebrafish, however, possesses two protocadherin loci that contain more than twice the number of genes as the coelacanth, but arranged only into α and γ clusters. To gain further insight into the evolutionary history of protocadherin clusters, we have sequenced and analyzed protocadherin clusters from the compact genome of the pufferfish, *Fugu rubripes*.

**Results:**

Fugu contains two unlinked protocadherin loci, *Pcdh1 *and *Pcdh2*, that collectively consist of at least 77 genes. The fugu *Pcdh1 *locus has been subject to extensive degeneration, resulting in the complete loss of *Pcdh1γ *cluster. The fugu *Pcdh *genes have undergone lineage-specific regional gene conversion processes that have resulted in a remarkable regional sequence homogenization among paralogs in the same subcluster. Phylogenetic analyses show that most protocadherin genes are orthologous between fugu and zebrafish either individually or as paralog groups. Based on the inferred phylogenetic relationships of fugu and zebrafish genes, we have reconstructed the evolutionary history of protocadherin clusters in the teleost fish lineage.

**Conclusion:**

Our results demonstrate the exceptional evolutionary dynamism of protocadherin genes in vertebrates in general, and in teleost fishes in particular. Besides the 'fish-specific' whole genome duplication, the evolution of protocadherin genes in teleost fishes is influenced by lineage-specific gene losses, tandem gene duplications and regional sequence homogenization. The dynamic protocadherin clusters might have led to the diversification of neural circuitry among teleosts, and contributed to the behavioral and physiological diversity of teleosts.

## Background

A long-standing mystery facing neurobiologists is the molecular mechanism underlying the highly-diversified neural network in vertebrate brains [1]. The discovery of three closely-linked protocadherin (*Pcdh*) clusters in mammalian genomes has led to an intriguing speculation that these genes may provide a profound molecular code for specifying neuron-neuron connections in the central nervous system [2-4]. Each of the three clusters, designated *Pcdh α*, *β*, and *γ *clusters, contains different numbers of large (~2.4 kb each) 'variable' exons. Each of these exons encodes an extracellular domain comprising six repeats of calcium-binding ectodomain (EC1-EC6), a transmembrane domain and a short cytoplasmic segment. The 3' ends of the α and γ (but not the β) clusters contain three 'constant' exons each, that are alternatively spliced to individual variable exons in their respective clusters. The constant exons encode the main part of the cytoplasmic domain shared by all members in the same cluster [2,3]. In many ways, this type of genomic organization resembles the immunoglobulin and T-cell receptor gene loci, which are widely known for their ability to generate a remarkably diverse repertoire of antigen recognizing molecules. *Pcdh *genes are expressed mainly in the neurons, and their proteins are highly enriched on synaptic membranes [2,5,6]. The transcription of *Pcdh *genes is controlled by individual promoters located adjacent to each variable exon [7,8], which contribute to the differential expression patterns of individual *Pcdh *genes in the central nervous system [5]. The *Pcdh *genes also appear to be under a higher order of complex regulation since their expression seems to be allele-selective [9], and individual neurons, even of the same kind, express an overlapping but distinct combination of *Pcdh *genes [7,8]. More recently, two long-range *cis*-regulatory elements in *Pcdhα *cluster have been identified and proposed to underlie the monoallelic expression of the *Pcdh *genes [10]. Taken together, these features of *Pcdh *genes indeed suggest that they have the potential to play a fundamental role in establishing neural diversity in the brain.

The *Pcdh *clusters are essentially a vertebrate innovation that accompanied the emergence of the neural tube and the elaborate central nervous system. No such *Pcdh *cluster has been identified in invertebrate genomes [11]. Mammals contain a single *Pcdh *locus consisting of about 60 genes [3,6,12-15]. The lobe-finned fish, coelacanth, which is believed to be a forerunner of tetrapods, also contains a single *Pcdh *locus organized into α, β, and γ clusters similar to mammals, with a total of 49 genes [16]. In contrast, the teleost fish, zebrafish, contains two unlinked *Pcdh *loci (*DrPcdh1 *and *DrPcdh2*), presumably due to the 'fish-specific' genome duplication [17,18]. The zebrafish genes in each locus are organized into only α and γ clusters. The two loci collectively contain at least 107 genes. The massive expansion of *Pcdh *genes in zebrafish has been attributed to lineage-specific expansion of individual genes in some *Pcdh *clusters [19,20]. Interestingly, the zebrafish *Pcdh *genes have experienced concerted evolution through adaptive selection and gene conversion [20]. Thus, the structure and organization of *Pcdh *clusters in zebrafish is quite divergent from that in lobe-finned fish and mammals. It has been speculated that the differences in the complement of *Pcdh*s in zebrafish and mammals could be related to the anatomical differences of their brains [20]. However, it is not known whether the organization of *Pcdh *clusters in zebrafish is typical of all teleost fishes or unique to the zebrafish lineage. Teleosts are the largest and most successful group of vertebrates. The extant teleosts include almost the same number of species as all other living vertebrate species combined. Teleost fishes also exhibit wide diversity in their habitat, morphology, behavior, physiology and adaptations [21]. Given the possible function of protocadherins in the formation of neural complexity, it would be of interest to characterize *Pcdh *clusters from diverse groups of teleost fishes.

In this study, we report the sequencing and comparative analysis of *Pcdh *clusters in the pufferfish, *Fugu rubripes*. Pufferfishes are unique in having the smallest genome among vertebrates. The reduction in the genome size of pufferfish is attributed to a paucity of repetitive sequences and short intergenic regions and introns. At 400 Mb, the fugu genome is one-eighth the human genome and one-quarter the size of the zebrafish genome. A 'draft' sequence of the fugu genome was completed in 2002 purely by the whole-genome shotgun sequencing strategy [22]. However, we found that most of *Pcdh *genes were misassembled in the 'draft' genome sequence, most likely due to the presence of a highly similar 3' region (identity >99% across about 750 bp) shared by all the variable exons in the same paralog subcluster (see Results and discussion below). We therefore sequenced overlapping cosmid and BAC clones and meticulously assembled the complete sequence for the *Pcdh *loci. Our results show that there are two unlinked *Pcdh *loci in fugu, similar to zebrafish, and they contain at least 77 genes. The *Pcdh1 *locus in fugu has undergone an extensive degeneration, resulting in the complete loss of the γ cluster. Based on the inferred evolutionary relationships of fugu and zebrafish *Pcdh *genes, we were able to reconstruct the two *Pcdh *loci in the common ancestor of fugu and zebrafish and the ancestral single *Pcdh *locus in the teleost fish lineage prior to the 'fish-specific' whole-genome duplication. Our data indicate that *Pcdh *clusters in teleost fishes have undergone extensive diversification largely through lineage-specific degeneration, tandem gene duplication, and gene conversion.

## Results and discussion

### Two unlinked protocadherin loci in fugu

**Figure 1:**
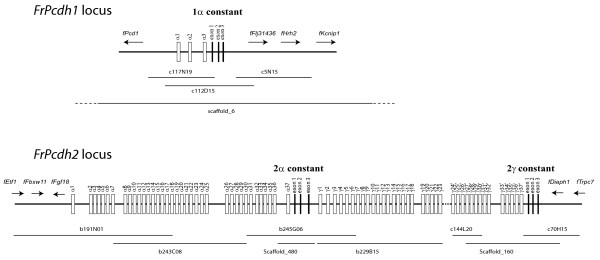
**Genomic organization of the two fugu protocadherin loci (*FrPcdh1 *and *FrPcdh2*)**. White boxes represent variable exons whereas solid bars at the end of each cluster represent the constant exons. The dotted line in *FrPcdh2γ *cluster represents a gap in the sequence. The IDs and position of the BAC and cosmid clones, as well as the relevant scaffolds, are shown below the gene clusters. The names of variable exons after the gap carry a prime sign (*γ24' *to *γ37'*) to indicate that the numbers may not reflect their actual positions in the locus. The non-*Pcdh *flanking genes and their orientation at each end of the locus are indicated by arrows. *fPcd1*: protocadherin 1 (a non-clustering protocadherin gene with multiple coding exons), *fFlj31436*: a homolog of human hypothetical protein *FLJ31436*, *fHrh2*: histamine receptor H2, *fKcnip1*: Kv channel interacting protein 1, *fEtf1*: eukaryotic translation termination factor 1, *fFbxw11*: F-box and WD-40 domain protein 1B, *fFgf18*: fibroblast growth factor 18, *fDiaph1*: diaphanous 1, *fTrpc7*: transient receptor potential cation channel, subfamily C, member 7.

We searched the fugu 'draft' genome assembly for scaffolds containing *Pcdh *genes by TBLASTN using the human *Pcdh *protein sequences as the query. Altogether we identified about 70 scaffolds with high similarity (*p *value < 10^-10^) to *Pcdh *sequences. A closer inspection of the scaffold sequences showed that most of them were either misassembled *Pcdh *cluster sequences, due to the presence of a stretch of nearly identical sequences across about 750 bp shared by multiple *Pcdh *variable exons, or contained non-clustering *Pcdh *genes. Only three scaffolds (scaffold_6, scaffold_160 and scaffold_480) contain reliably-assembled sequences equivalent to the *Pcdh *constant regions. Detailed examination of scaffold_6 (~3.4 Mb long) revealed that this scaffold contains a small *Pcdhα *cluster, consisting of three variable exons followed by three constant exons, but no *Pcdhγ *genes (Fig 1). These genes are flanked by several non-*Pcdh *genes, indicating that this is a complete *Pcdh *locus (Fig 1). To verify this, we have identified three overlapping cosmid clones that span this region (Fig 1). The sizes of these cosmid clones, as inferred by mapping their end sequences to scaffold_6 sequence, range from 40.2 to 48.7 kb. These are typical sizes of cosmid clones and thus indicate that there are no large scale deletions or insertions at the *Pcdh *locus on the assembled scaffold_6 sequence. To confirm this, we analyzed cosmid clone c112D15, whose sequence spans the entire *Pcdh1 *locus, by restriction mapping using three enzymes (EcoRI, HindIII and EcoRV) and found that it contains 46 kb insert and its restriction map exactly matches that predicted from scaffold_6 sequences (data not shown). This confirms that the *Pcdh *cluster on scaffold_6 represents a complete fugu *Pcdh *locus, and contains only three *Pcdhα *genes and no *Pcdhγ *genes. Phylogenetic analyses of the variable (see below) and constant (data not shown) exons showed that these genes are orthologous to zebrafish *DrPcdh1α *genes. We thus designated this *Pcdh *locus as *FrPcdh1 *(Fig 1). We filled gaps in the *Pcdh *constant region in scaffold_480 and scaffold_160 by PCR using fugu genomic DNA as a template and extended the sequences by identifying and sequencing overlapping cosmid or BAC clones (see Methods). This approach resulted in two contiguous sequences of length 331 kb and 86 kb.

The larger contig includes a complete *Pcdhα *cluster containing 37 variable exons and three constant exons, followed by the first 23 variable exons of a *Pcdhγ *cluster (Fig 1). We identified three non-*Pcdh *genes, *fEtf1*, *fFbxw11 *and *fFgf18 *upstream of the *Pcdhα *cluster indicating that the *α *cluster on this contig is complete (Fig 1). The shorter contig contains 14 variable exons and three constant exons of a *Pcdhγ *cluster, followed by two non-*Pcdh *genes, *fDiaph1 *and *fTrpc7 *(Fig 1). RT-PCR with forward primers corresponding to variable exons of the *Pcdhγ *cluster of the larger contig and a reverse primer for the constant region of *Pcdhγ *in the shorter contig (data not shown) showed that the two contigs belong to the same locus. We designate this locus as *FrPcdh2 *locus (Fig 1). We were unable to fill the gap between the two contigs due to the lack of a genomic clone spanning the two contigs. Attempts to fill the gap by long-template PCR using fugu genomic DNA as a template also failed, presumably due to the large size of the gap between the two contigs. The *FrPcdh2 *locus contains 37 α variable exons and at least 37 γ variable exons. The exons downstream of the gap have been numbered with a prime sign (24' to 37') to indicate that the numbers may not represent their actual positions in the cluster. Thus, fugu possesses two unlinked *Pcdh *loci, *Pcdh1 *and *Pcdh2*.

The two *Pcdh *loci in fugu apparently resulted from segmental duplication from an ancestral *Pcdh *cluster. Phylogenetic analyses using constant (data not shown) and variable (see below) regions of fugu and zebrafish *Pcdh *genes show that the two *Pcdh *loci in fugu are orthologous to the duplicate zebrafish *Pcdh *loci, respectively, indicating that the locus duplication took place before the divergence of the two lineages. This duplication is most likely the result of the "fish-specific' whole genome duplication event that occurred early during the evolution of ray-finned fishes [17,18]. Like the two zebrafish *Pcdh *loci, both fugu *Pcdh *loci lack *β *cluster genes. The presence of a *β *cluster in the lobe-finned fish and tetrapods, and its absence in fugu and zebrafish suggest that the *β *cluster either evolved in the lineage that led to the lobe-finned fish and tetrapods, or was already present in the common ancestor of these vertebrates and was subsequently lost in the teleost lineage before the divergence of the fugu and zebrafish lineages.

The two *Pcdh *loci in the fugu and zebrafish show significant differences in their gene content and organization. For instance, *FrPcdh1 *cluster is highly degenerate compared to the zebrafish *Pcdh1*; as a result, it contains only three α genes compared to ten α genes in zebrafish *Pcdh1 *cluster [12,19,20]. More strikingly, the fugu *Pcdh1 *locus completely lacks a γ cluster (Fig 1), whereas the zebrafish *Pcdh1 *locus contains a γ cluster with at least 28 genes [12,19,20]. Thus, unlike the zebrafish genome which contains two *Pcdhγ *clusters, fugu genome contains a single *Pcdhγ *cluster that is located in the *Pcdh2 *locus. The whole-genome sequence of a second pufferfish, *Tetraodon nigroviridis*, has recently been completed [23]. To determine whether *Tetraodon *contains a single *Pcdhγ *cluster like the fugu, we performed a BLASTX search of the *Tetraodon *genome database and discovered that *Tetraodon *also contains two sets of α constant exons belonging to two putative *Pcdh *clusters but only a single set of γ constant exons similar to fugu. Thus the second copy of *Pcdhγ *cluster associated with the *Pcdh1 *locus seems to have been lost before the divergence of the fugu and *Tetraodon*. The highly degenerate nature of the *Pcdh1 *locus in pufferfishes is consistent with the trend of pufferfish genome towards compaction. The complete loss of the second copy of *Pcdhγ *cluster in pufferfish suggests that these *Pcdhγ *genes may be redundant. However, we cannot rule out the possibility that the loss of this cluster in pufferfishes might have an effect on their phenotype with regard to the structure and function of the central nervous system.

### Phylogenetic relationships of fugu, zebrafish and coelacanth protocadherin genes

**Figure 2:**
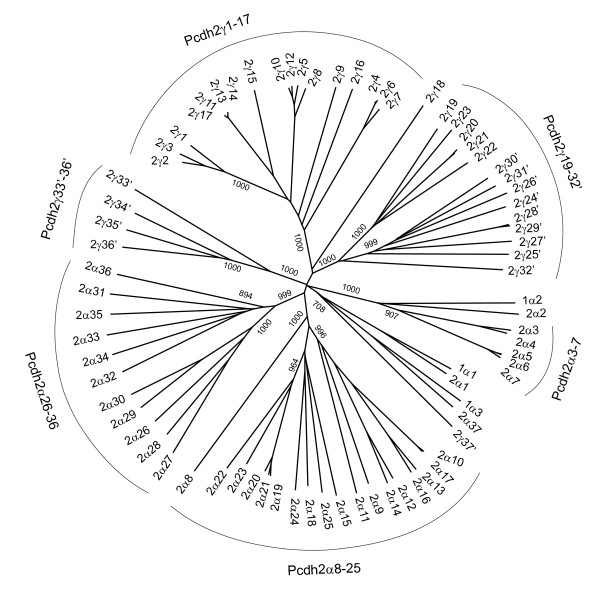
**Phylogenetic relationships of fugu protocadherin genes**. Protein sequences for the EC1-EC3 ectodomain region of fugu *Pcdh *α and γ genes were aligned by ClustalX. The phylogenetic tree was constructed by the Neighbor-joining method based on sequence distance matrix. The tree is unrooted. Numbers at the nodes are bootstrap values of 1000 replicates. Only bootstrap values above 500 in the major branches are shown.

The *FrPcdh2 *locus contains 37 *α *genes and at least 37 *γ *genes, as compared to 38 *α *genes and at least 31 *γ *genes in the zebrafish *Pcdh2 *locus [16,19,20]. In order to determine the phylogenetic relationships of these genes and to trace the evolutionary history of *Pcdh *clusters in teleosts, we performed phylogenetic analyses using the Neighbor-joining method. We used only EC1-EC3 sequences, instead of the entire ectodomain region (EC1-EC6) for the analyses, because the C-terminal ectodomain (EC4-EC6) of some fugu and zebrafish genes have undergone extensive regional sequence homogenization due to repeated gene conversion events (see below), and using such homogenized regions would bias the tree and the inferred relationships. We first determined the relationships among fugu *Pcdh1 *and *Pcdh2 *genes (Fig 2). The topology of the gene tree shows that *FrPcdh2α *cluster is mainly comprised of three major paralog groups, *FrPcdh2α3–7*, *FrPcdh2α8–25 *and *FrPcdh2α26–36*. The three α genes in the *Pcdh1 *cluster, *FrPcdh1α1-α3*, are the inter-locus paralog of *FrPcdh2α1*, *α2 *and *α37 *of the *Pcdh2 *locus, respectively. *FrPcdh2γ *cluster also consists of three large paralog groups, *FrPcdh2γ1–17*, *FrPcdh2γ19–32' *and *FrPcdh2γ 33'-36'*. In addition, *Pcdhγ *cluster also contains two individual genes, *FrPcdh2γ18 *and *FrPcdh2γ37'*, that seem to be distantly related to the other genes in the cluster, suggesting they are generated from ancient gene duplications. Interestingly, *FrPcdh2γ37' *appears to be more closely related to the *FrPcdh1α3 *and *FrPcdh2α37 *in the *Pcdhα *cluster (Fig 2). Notably, such a phylogenetic relationship between *Pcdhα *and *Pcdhγ *clusters is also evident in mammalian *Pcdh *clusters [3,12,13]. The two genes, c1 and c2, at the end of mammalian *Pcdhα *cluster are shown to be evolutionarily closer to the last three genes, c3-c5, of the *Pcdhγ *cluster than any other genes in the *Pcdhα *cluster [3]. Interestingly, phylogenetic analyses show that *FrPcdh1α3*, *FrPcdh2α37 *and *FrPcdh2γ37 *indeed belong to the mammalian c1-c5 gene group (see below). The remarkable conservation of these genes suggests that they may play an important role in protocadherin functions in all vertebrates. The overall structure of fugu *Pcdh *cluster gene tree is highly similar to that of zebrafish, which also contains three large paralog groups each of *Pcdhα *and *γ *clusters [12].

**Figure 3:**
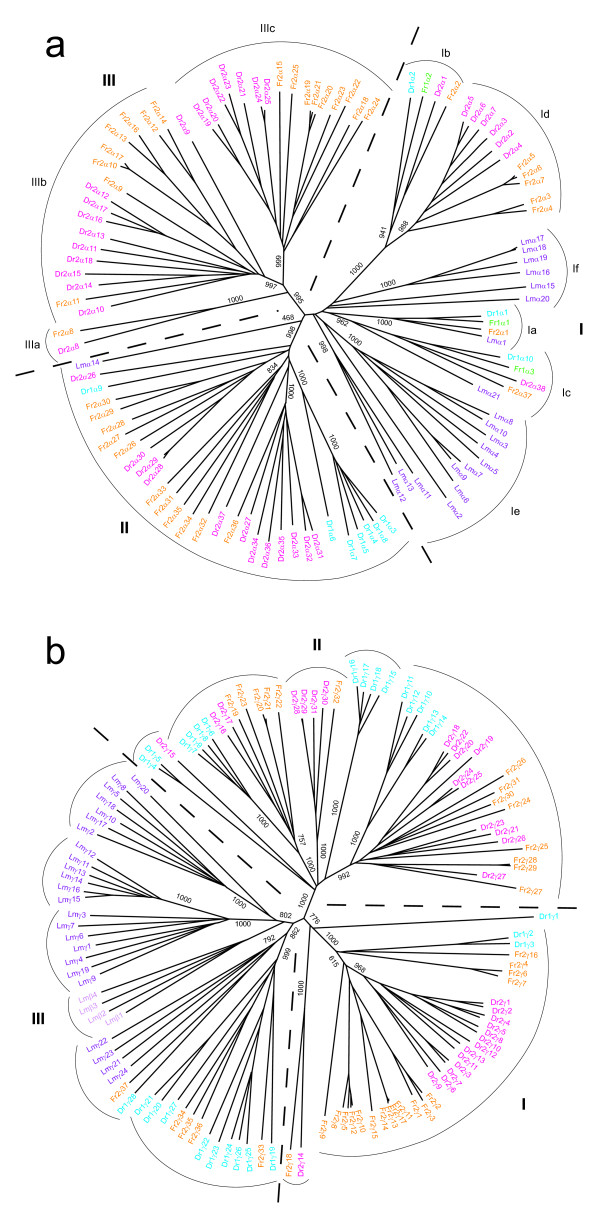
**Phylogenetic analyses of fugu, zebrafish and coelacanth protocadherin genes**. Phylogenetic trees for *Pcdhα *(**a**) and *Pcdhγ *(**b**) clusters. Protein sequences of the EC1-EC3 ectodomain region were aligned by ClustalX and phylogenetic trees were built by the Neighbor-joining method based on sequence distance matrix. Numbers at the nodes are bootstrap values of 1000 replicates. Only bootstrap values above 500 at the major branches are shown. The trees are unrooted. Genes in individual *Pcdhα *or *Pcdhγ *clusters are labeled by the same color.

To explore the orthology of fugu and zebrafish *Pcdh *genes and their phylogenetic relationships with *Pcdh *genes from other vertebrate groups, we next performed phylogenetic analyses of fugu and zebrafish *Pcdh *genes together with *Pcdh *genes from coelacanth. Coelacanth was selected as a representative of lobe-finned fish and tetrapod lineages since the single *Pcdh *cluster in coelacanth is likely to be the closest to the ancestral *Pcdh *locus of the two teleost lineages. We analyzed *Pcdhα *(Fig 3a) and *Pcdhγ *(Fig 3b) clusters separately.

As shown in Fig 3a, *Pcdhα *genes of fugu, zebrafish and coelacanth comprise three large paralog/ortholog groups. The first group (group I in Fig 3a) contains genes localized at the two ends of fugu and zebrafish *Pcdhα *clusters, including fugu *FrPcdh1α1–3*, *FrPcdh2α1–7*, *FrPcdh2α37 *and zebrafish *DrPcdh1α1–2*, *DrPcdh1α10*, *DrPcdh2α1–7*, *DrPcdh2α38*, besides all but one of the genes (*LmPcdhα14*) in the coelacanth *Pcdhα *cluster. These fugu and zebrafish genes are further divided into four subgroups. The first subgroup (Ia in Fig 3a) consists of two fugu inter-locus paralogs, *FrPcdh1α1 *and *FrPcdh2α1*, zebrafish *DrPcdh1α1 *and *LmPcdhα1*. The second subgroup (Ib in Fig 3a) is comprised of two fugu inter-locus paralogs, *FrPcdh1α2*, *FrPcdh2α2 *and their zebrafish orthologs, *DrPcdh1α2 *and *DrPcdh2α1*. The third subgroup (Ic in Fig 3a) contains fugu *FrPcdh1α3 *and *FrPcdh2α37 *and their zebrafish orthologs *DrPcdh1α10 *and *DrPcdh2α38*, as well as the coelacanth ortholog, *LmPcdha21*. An interesting feature of these *Pcdh *genes is that they seem to be resistant to gene duplication. In spite of the heavy turnover of genes in their neighborhood (see below), they have been conserved as single-copy genes throughout the evolution of these vertebrates. This suggests that they may play a fundamental role in the central nervous system. The fourth subgroup (Id in Fig 3a) contains fugu *FrPcdh2α3–7 *and zebrafish *DrPcdh2α2–7*. No direct orthologous relationship can be identified between individual genes in this subgroup; instead, *FrPcdh2α3–7 *as a paralog group seems to be orthologous to *DrPcdh2α5–7*. This type of phylogenetic relationship indicates that subsequent to the divergence of the two species, the ancestral paralogs have undergone independent lineage-specific gene duplications, giving rise to a multi-gene paralog group in each species. This phylogenetic tree also suggests that the subgroup Ia and Ic are derived from a common ancestor, while subgroup Ib and Id share a common ancestor. Except *LmPcdhα1 *and *LmPcdhα21*, other coelacanth genes in this group do not show any direct orthology to fugu and zebrafish genes, suggesting that these genes are either specific to lobe-finned fish and tetrapods or have been lost from the teleost fish lineage.

The second paralog/ortholog group (group II in Fig 3a) in the *Pcdhα *phylogenetic tree comprises fugu *FrPcdh2α26–36*, zebrafish *DrPcdh1α3–9*, *DrPcdh2α26–37*, and a single coelacanth gene, *LmPcdhα14*. The subtrees of this group show that a subset of genes in the zebrafish *Pcdh1α *locus, the *DrPcdh1α(3–5,7–8)*, are generated from an ancestral paralog of *DrPcdh1α6 *through multiple gene duplication events in the zebrafish lineage. No fugu ortholog for zebrafish *DrPcdh1α3-*9 genes is found in *FrPcdh1 *locus, presumably due to the independent loss of this paralog group of genes in fugu. On the other hand, *FrPcdh2α31–35 *appear to be derived from a single common ancestor through lineage-specific duplications in fugu. A single fugu gene, *FrPcdh2α36*, seems to share a common ancestor with a cluster of zebrafish genes, *DrPcdh2α(27,31–36)*, indicating that while the fugu gene was retained as single-copy, the zebrafish gene has undergone multiple duplications. The fourth subset of genes in this paralog/ortholog group consists of multiple fugu and zebrafish genes including *FrPcdh2α26–30*, *DrPcdh2α(26,28–30) *and *DrPcdh1α9*. However, the orthologous relationship between these subsets of genes cannot be inferred with confidence since the bootstrap values at their branch nodes are rather low (< 200). As these paralog/ortholog group genes are closely related and are clearly segregated from other fugu and zebrafish *Pcdh *paralog/ortholog group genes, we consider the whole group as one large paralog/ortholog group. The evolution of such paralog/ortholog groups is likely to have involved many rounds of lineage-specific gene duplication and degeneration. It appears that *LmPcdhα14 *is a distant ortholog of this group (Fig 3a). Interestingly, this coelacanth gene also shares common ancestry with the entire mammalian α cluster (except the c1 and c2 genes) [16], suggesting that this paralog/ortholog group of fugu and zebrafish genes is perhaps orthologous to the entire mammalian *Pcdhα *cluster.

The third paralog/ortholog group of *Pcdhα *genes (group III in Fig. 3a) seems to be teleost-specific, containing only fugu *FrPcdh2α8–25 *and zebrafish *DrPcdh2α8–25*. These genes can further be divided into three subgroups. The first subgroup (IIIa in Fig 3a) contains fugu *FrPcdh2α8 *and its zebrafish ortholog *DrPcdh2α8*, whereas the other two subgroups (IIIb and IIIc in Fig 3a) that contain multiple fugu and zebrafish paralogs do not exhibit any manifest individual orthologous relationships. However, it is clear that fugu *FrPcdh2α (15,18–25) *and *FrPcdh2α (9–14,16–17) *as paralog subgroups are orthologous to zebrafish *DrPcdh2α19–25 *and *DrPcdh2α9–18*, respectively.

Similar to *Pcdhα *genes, the *Pcdhγ *genes also form three large paralog/ortholog groups (Fig 3b). Orthology between multi-gene groups (between a single gene and a subset of genes and between two subsets of genes in two species) seems also to be a common feature of the *Pcdh1γ *cluster. For example, the fugu *FrPcdh2γ32' *(group II in Fig 3b) is apparently orthologous to the entire zebrafish paralog group *DrPcdh2γ28–31*, whereas the fugu *FrPcdh2γ1–17 *(group I in Fig 3b) as a paralog group is orthologous to a zebrafish *DrPcdh2γ1–13*. Additionally, orthology between two individual genes from two species is also observed in the *Pcdhγ *cluster. For instance, fugu *FrPcdh2γ18 *(group I in Fig 3b) is clearly an ortholog of zebrafish *DrPcdh2γ14*. Consistent with the previous study [16], the phylogenetic tree for *Pcdhγ *cluster also revealed that coelacanth *Pcdhγ *genes comprise five paralog groups (group III in Fig 3b), of which four, *LmPcdhγ(1,3–4,7,9,19)*, *LmPcdhγ11–16*, *LmPcdhγ(2,5,8,17–18,20) *and *LmPcdhβ1–4 *are closely related to each other, whereas the fifth group, *LmPcdhγ21–24*, is more closely related to fugu *FrPcdh2γ37' *and zebrafish *DrPcdh1γ28 *(Fig 3b). Such a phylogenetic relationship suggests that a massive expansion of *Pcdhγ *genes has occurred in the coelacanth lineage subsequent to the divergence of these species.

Orthology between an individual gene in one species and a group of genes in another and between groups of genes in two species rather than between individual genes is a characteristic of multigene families which have experienced continuous events of lineage-specific gene duplications and losses. *Pcdh *cluster is a typical example of such a dynamic cluster of genes in vertebrates. The *Pcdh *clusters from fugu and zebrafish include instances of orthology between a single fugu gene and a group of paralogous zebrafish genes (*e.g*., *FrPcdh2γ32' *and *DrPcdh2γ28–31*) and between entire paralog groups of fugu and zebrafish genes (*e.g*., *FrPcdh2γ1–17 *and *DrPcdh2γ1–13*). These types of phylogenetic relationships among *Pcdh *genes in fugu and zebrafish illustrate the exceptionally dynamic evolutionary changes at the *Pcdh *loci in the teleost fish lineage following the 'fish-specific' whole genome duplication event. Although the single *Pcdh *cluster in mammals and the coelacanth have experienced gene duplications and losses, the extent of turnover is much lower than that in the fugu and zebrafish. Such variations in the complement of *Pcdh *genes show that *Pcdh *clusters are much more dynamic in teleost fishes than in mammals and lobe-finned fishes. Since teleost fishes are the most species-rich and most diverse group of vertebrates, it is likely that the evolutionarily dynamic *Pcdh *clusters in teleosts might have contributed to morphological and behavioral diversity of teleost fishes.

### Regional gene conversion in fugu protocadherin locus

A striking feature of fugu *Pcdh *cluster genes observed during the assembly of cosmid and BAC sequences is the highly similar 3' region of variable exons (identity >99%) shared by multiple paralogs. These paralogs, which can be differentiated by their divergent 5' sequences, are generally located at close proximity on the chromosome and segregate into subclusters. We have identified three such paralog subclusters in each of the fugu *Pcdh2α *(*FrPcdh2α2–7*,* FrPcdh2α8–25 *and *FrPcdh2α26–36*) and *Pcdh2γ *(*FrPcdh2γ1–17*, *FrPcdh2γ19–32' *and *FrPcdh2γ33'-36'*) clusters, respectively. To investigate the extent of sequence similarity and differences in the 5' and 3' regions of these genes, we aligned protein and nucleotide sequences of individual paralog subclusters and calculated their pair-wise amino acid and nucleotide sequence identities based on the multiple alignment. While the sequence identity of the less similar upstream region ranges from 60 to 80% at both amino acid and nucleotide levels, the 3' sequences are nearly 100% identical among members in each subcluster (Table 1). The two distinct regions are separated by a discrete boundary located at the coding sequence for domains EC4 or EC5 in different subclusters (Table 1). Because purifying selection for protein function does not act on synonymous sites, the astonishingly high sequence similarity at the nucleotide level is thus unlikely to be due to a greater functional constraint on the protein sequence. Instead, such homogenized sequences could have arisen from repeated regional gene conversion events. There is evidence that tandem gene arrays tend to embark on gene conversion that leads to sequence homogenization among paralogs [24]. Indeed, zebrafish and human *Pcdh *paralogs have been shown to have undergone frequent gene conversions, resulting in substantial sequence homogenization among paralogs [12,20,25]. However, gene conversions do not seem to be an inherent characteristic of all *Pcdh *clusters, because no gene conversion signatures have been uncovered at the coelacanth *Pcdh *locus [16]. To determine whether the high similarity regions shared by fugu paralogs are generated through gene conversion events, we compared the GC content at codon third positions (GC3) between the 5' low similarity and the 3' high similarity regions among paralogs in each group. It is known that repeated gene conversions usually cause an increase of GC3 in converted regions [26,27]. The GC3 in 3' regions of all the paralog groups is above 50%, and is significantly higher than that for their corresponding 5' regions (Table 2), indicating that these high similarity regions are indeed the result of gene conversion events.

**Table 1:** Paralog sequence similarity of fugu protocadherin subclusters

	5' low homology region			3' high homology region		
		
Paralog subclusters^a^	Pcdh protein sequence^b^	Amino acid identity^c ^(%)	Nucleotide identity^d ^(%)	Pcdh protein sequence^b^	Amino acid identity^c ^(%)	Nucleotide identity^d ^(%)
FrPcdh2α2–7	1–331	80.0 ± 13.4	79.0 ± 13.9	332–777	99.3 ± 0.3	99.3 ± 0.2
FrPcdh2α8–25	1–507	67.6 ± 10.8	69.9 ± 9.9	508–759	99.4 ± 0.3	99.1 ± 0.2
FrPcdh2α26–36	1–487	57.5 ± 6.6	60.7 ± 5.1	488–770	99.4 ± 0.3	99.4 ± 0.3
						
FrPcdh2γ1–17	1–356	70.0 ± 10.8	72.7 ± 10.0	357–764	99.6 ± 0.3	99.3 ± 0.3
FrPcdh2γ19–32'	1–507	66.4 ± 9.0	68.2 ± 8.5	508–781	99.3 ± 0.3	99.3 ± 0.2
FrPcdh2γ33'-36'	1–449	66.5 ± 9.5	68.7 ± 7.3	450–792	99.3 ± 0.6	99.6 ± 0.3

^a ^The fugu *Pcdh *sequences used for this analysis do not include the coding sequences for the signal peptide.

^b ^The numbers refer to the consensus of the amino acid sequence alignment without the signal peptide sequence

^c ^The amino acid sequence identity is calculated by average of the pair-wise comparison of paralog members in the same subcluster and expressed as mean ± standard deviation.

^d ^Nucleotide sequences were aligned according to the amino acid alignment and the percentage identity is calculated by average of the pair-wise comparison and expressed as mean ± standard deviation.

**Table 2:** GC3 of fugu paralog subcluster protocadherin sequences

Paralog subclusters	GC3 (%)		
	
	5' low homology region^a^	3' high homology region^a^	*p *Value^b^
FrPcdh2α2–7	41.3 ± 7.2	61.2 ± 0.3	2.78 × 10^-4^
FrPcdh2α9–25	38.4 ± 4.3	54.4 ± 0.5	2.68 × 10^-16^
FrPcdh2α26–36	45.0 ± 2.2	60.7 ± 0.7	1.32 × 10^-15^
			
FrPcdh2γ1–17	43.6 ± 2.6	56.3 ± 0.4	2.40 × 10^-19^
FrPcdh2γ19–32'	41.5 ± 3.4	58.5 ± 0.6	3.28 × 10^-15^
FrPcdh2γ33'-36'	40.1 ± 1.2	68.8 ± 0.3	6.35 × 10^-9^

^a ^GC3 is calculated as the average percentage of the GC content at the codon third-position in each paralog subcluster and expressed as mean ± standard deviation.

^b ^The statistical analysis was conducted using *Student's t-test*.

Pcdhs have been proposed to provide molecular diversities for neuron-neuron connections through the combinatorial interaction of protocadherin proteins. For classical cadherins, the *trans*-homophilic interaction (*i.e*. the interaction between cells) is mainly mediated by the EC1 domain [28]. Although yet to be demonstrated experimentally, it is generally believed that Pcdhs also engage in a similar form of homophilic interaction as the classic cadherins. However, unlike classic cadherins which contain five ectodomains in their extracellular region, the extracellular region of Pcdhs contains six ectodomains. It is possible that the molecular diversifying signals of Pcdhs in fugu are encoded by the extracellular EC1-EC3 domains since this region is more divergent among individual Pcdhs as compared to the highly homologous C-terminal extracellular domains. This is consistent with the observation that the EC2 and EC3 domains of zebrafish and mammalian Pcdhs seldom undergo sequence homogenization processes and thus provide the most diversifying signals for the molecules [20]. Interestingly, it has been shown recently that EC2 and EC3 of mammalian Pcdhs undergo diversity-enhancing positive diversifying selection [12]. Collectively, these observations imply that the N-terminal ectodomains of Pcdhs play a crucial role in mediating neuronal connections in the brain. Furthermore, in contrast to the virtually 100% identical C-terminal sequences of paralogs in the same fugu subclusters, the converted regions are highly divergent between subclusters. The consensus sequences for the converted regions between different subclusters of *Pcdh2α *and *Pcdh2γ *exhibit on average only 37.7% and 38.9% identities, respectively. This implies that the converted regions in different subclusters may have undergone adaptive selection and acquired diverse functions specific to each subcluster. In contrast to fugu *Pcdh2 *cluster genes, the *Pcdh1 *cluster genes do not contain any signature for gene conversion.

### Reconstruction of protocadherin clusters in ancestral fish lineage

**Figure 4:**
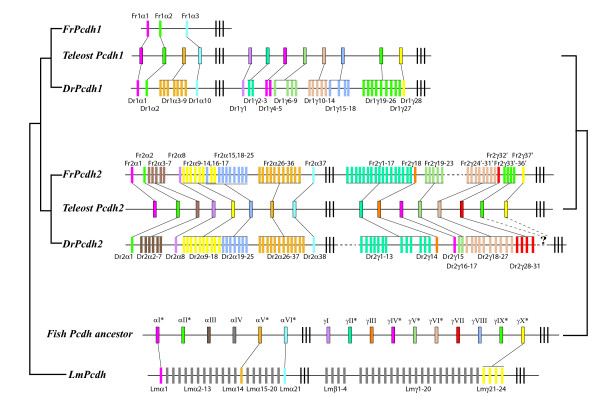
**Comparison of the fugu (*FrPcdh1 *and *FrPcdh2*), zebrafish (*DrPcdh1 *and *DrPcdh2*)and coelacanth (*LmPcdh*) protocadherin clusters**. Variable exons in each paralog group are shown in different colors. Orthologs between fugu and zebrafish as well as the inter-locus paralogs between the two *Pcdh *loci in fugu or zebrafish are shown in the same colors. '*Teleost Pcdh1*' and '*Teleost Pcdh2*' are the *Pcdh *clusters predicted in the common ancestor of fugu and zebrafish, and '*Fish Pcdh ancestor*' is the single *Pcdh *cluster predicted in the ray-finned fish prior to the 'fish-specific' whole genome duplication. The corresponding exons in the '*Fish Pcdh ancestor*' and the inter-locus paralogs between '*Teleost Pcdh1*' and '*Teleost Pcdh2*' are shown in the same color except the 'α*IV*', which represents a common ancestor for fugu *FrPcdh2α8–25 *and zebrafish *DrPcdh2α8–25*. Among the exons predicted in the '*Fish Pcdh ancestor*', those present in the *Pcdh *loci of both fugu and zebrafish are labeled with an asterisk.

Based on the inferred phylogenetic relationships of fugu, zebrafish and coelacanth *Pcdh *genes, we have reconstructed models of the duplicate teleost *Pcdh *clusters in the common ancestor of fugu and zebrafish, and the single *Pcdh *cluster in the fish lineage prior to the 'fish-specific' whole genome duplication event (Fig 4). These models illustrate the dynamic nature of the *Pcdh *locus in vertebrates. The *Pcdh *loci in teleost fishes and the coelacanth have repeatedly experienced lineage-specific gene losses and gene duplications. The lineage-specific tandem gene duplication is rather dramatic in the *Pcdh2 *locus of teleost fishes, giving rise to at least 74 and 69 genes in fugu and zebrafish respectively, compared to 49 genes in the coelacanth. According to our model, the single *Pcdh *cluster in the fish lineage prior to the whole genome duplication contained at least six *Pcdhα *paralog groups (*αI *to *αVI*) and ten *Pcdhγ *paralog groups (*γI *to *γX*) (Fig 4). In contrast, coelacanth *Pcdh *cluster contains orthologs for only three of these fish *Pcdhα *genes and one of the fish *Pcdhγ *genes. On the other hand, two of the coelacanth *Pcdhα *paralog groups (*Lmα2–13 *and *Lmα15–20*) and one *Pcdh *paralog group containing *Lmβ1–4 *and *Lmγ1–20 *have no apparent orthologs in the ancestral fish *Pcdh *cluster. These comparisons show that the *Pcdh *loci have been subject to dynamic changes since the divergence of the lobe-finned fish and ray-finned fish lineages and have been continuously undergoing lineage-specific degeneration and tandem duplications. Characterization of *Pcdh *cluster from a more basal vertebrate, such as a cartilaginous fish, should shed light on the ancestral state of *Pcdh *cluster(s) and help to reconstruct the evolutionary changes in the basal lobe-finned fishes and teleost fishes.

## Conclusion

We have identified two unlinked fugu *Pcdh *loci that collectively contain at least 77 *Pcdh *genes. The gene content of the two fugu *Pcdh *loci is quite different from that of the two *Pcdh *loci in zebrafish. We show that following the 'fish-specific' whole-genome duplication, regional sequence homogenization due to repeated lineage-specific gene conversion processes, secondary gene losses and tandem gene duplications are the major factors affecting the evolution of *Pcdh *clusters in teleosts. Based on phylogenetic analyses, we predict that there were at least six α and ten γ genes (or paralog groups) in the *Pcdh *locus of the ancestral fish genome prior to the whole-genome duplication event. Elucidating the origin and evolutionary dynamics of *Pcdh *clusters in different lineage of vertebrates is an important endeavor as it may help to uncover the molecular code for the complex central nervous system of vertebrates.

## Methods

### Sequencing and assembly of fugu *Pcdh *loci

To identify fugu *Pcdh *sequences in the fugu 'draft' genome, we performed TBLASTN search of fugu genome database using human protocadherin protein sequences as the query [22]. We identified about 70 scaffolds that showed similarity to Pcdh protein with an E-value of 10^-10 ^or less. Detailed examination of these scaffolds showed that most of the resulting scaffolds were misassembled due to the high sequence homology shared by multiple fugu *Pcdh *variable exons. Only three scaffolds, scaffold_6, scaffold_480 and scaffold_160, were found to contain large reliably-assembled sequences. Gaps within the relevant regions of these scaffolds were filled by PCR using fugu genomic DNA as a template. Scaffold_6 contained a complete *Pcdh *cluster flanked by non-*Pcdh *genes. We identified three overlapping cosmid clones that cover the *Pcdh*-containing region on scaffold_6. These include: c117N19, c112D15 and c5N15. For the other two scaffolds, scaffold_480 and scaffold_160, we used only the reliable *Pcdh*-containing sequences as the anchor sequence for identifying overlapping cosmid and BAC clones by BLASTN search of the cosmid or BAC end databases [22]. We first attempted to sequence these cosmid and BAC clones by shotgun method. However, since this resulted in piling up of many variable exons, we resorted to cloning and sequencing restriction enzyme-digested fragments to obtain contiguous sequences. The protocol for shotgun sequencing of cosmid and BAC clones comprised of shearing DNA by ultra-sonication followed by end-filling by *Klenow *treatment. The blunt-ended DNA fragments were resolved on an agarose gel and 2–3 kb fragments were isolated and subcloned into the EcoRV site of pBluescript SK vector. Plasmid inserts were sequenced from both ends using T3 and T7 primers and BigDye Terminator technology (Applied Biosystem). Sequence reads were then edited and assembled using SeqMan (Lasergene). The *Pcdh *variable exons and non-*Pcdh *genes were annotated based on the results of BLASTX search of the non-redundant protein database at NCBI [29] and GENSCAN predictions [30]. Sequences of fugu *Pcdh *clusters generated in this study have been submitted to GenBank under accession numbers DQ986917 and DQ986918. Human orthologs of the fugu non-*Pcdh *genes were identified by BLAT search of the human genome database at the UCSC genome browser [31].

### Phylogenetic analyses

The genomic sequences of zebrafish and coelacanth *Pcdh *clusters were retrieved from the GenBank [29]. The zebrafish *Pcdh *clusters were assembled from sequences of AC144823, AC144826, AC144828, AC146480, AL929558, AB075928, BX005294 and BX957322[12,16,19,20], whereas the coelacanth *Pcdh *clusters were assembled from sequences of AC150238, AC150284 and AC150308-AC150310[16]. Variable exons were identified by BLASTX searches. We used the N-terminal protocadherin ectodomain sequences (EC1-EC3) for constructing phylogenetic trees as this region is structurally homologous in all species, which gives rise to few gaps in the alignment and does not undergo gene conversion. The sequences of EC1-EC3 from various species were aligned by ClustalX algorithm [32]. Phylogenetic trees were constructed by the Neighbor-joining method based on sequence distance matrix, and the trees were drawn using NJplot [33]. The robustness of the tree was determined by bootstrap analysis of 1000 replicate sample sequences.

### Analysis for third position GC content

We used CODEML program in PAML package with default parameters to determine the GC content at third-position of codons [34]. The nucleotide sequence alignments were generated by RevTrans program using amino acid sequence alignment as templates [35].

## Authors' contributions

WPY and BV conceived the study. WPY, KY and VR designed the experimental strategy, performed the sequencing of fugu cosmid and BAC clones and contributed to acquisition and analyses of the experimental data. WPY conducted the phylogenetic and codon usage analyses. WPY and BV analyzed the data and wrote the manuscript. All authors read and approved the final manuscript.

## References

[B1] SPERRY RW (1963). Chemoaffinity in the orderly growth of nerve fiber patterns and connections. Proc Natl Acad Sci U S A.

[B2] Kohmura N, Senzaki K, Hamada S, Kai N, Yasuda R, Watanabe M, Ishii H, Yasuda M, Mishina M, Yagi T (1998). Diversity revealed by a novel family of cadherins expressed in neurons at a synaptic complex. Neuron.

[B3] Wu Q, Maniatis T (1999). A striking organization of a large family of human neural cadherin-like cell adhesion genes. Cell.

[B4] Shapiro L, Colman DR (1999). The diversity of cadherins and implications for a synaptic adhesive code in the CNS. Neuron.

[B5] Frank M, Ebert M, Shan W, Phillips GR, Arndt K, Colman DR, Kemler R (2005). Differential expression of individual gamma-protocadherins during mouse brain development. Mol Cell Neurosci.

[B6] Zou C, Huang W, Ying G, Wu Q (2007). Sequence analysis and expression mapping of the rat clustered protocadherin gene repertoires. Neuroscience.

[B7] Tasic B, Nabholz CE, Baldwin KK, Kim Y, Rueckert EH, Ribich SA, Cramer P, Wu Q, Axel R, Maniatis T (2002). Promoter choice determines splice site selection in protocadherin alpha and gamma pre-mRNA splicing. Mol Cell.

[B8] Wang X, Su H, Bradley A (2002). Molecular mechanisms governing Pcdh-gamma gene expression: evidence for a multiple promoter and cis-alternative splicing model. Genes Dev.

[B9] Esumi S, Kakazu N, Taguchi Y, Hirayama T, Sasaki A, Hirabayashi T, Koide T, Kitsukawa T, Hamada S, Yagi T (2005). Monoallelic yet combinatorial expression of variable exons of the protocadherin-alpha gene cluster in single neurons. Nat Genet.

[B10] Ribich S, Tasic B, Maniatis T (2006). Identification of long-range regulatory elements in the protocadherin-alpha gene cluster. Proc Natl Acad Sci U S A.

[B11] Hill E, Broadbent ID, Chothia C, Pettitt J (2001). Cadherin superfamily proteins in Caenorhabditis elegans and Drosophila melanogaster. J Mol Biol.

[B12] Wu Q (2005). Comparative genomics and diversifying selection of the clustered vertebrate protocadherin genes. Genetics.

[B13] Wu Q, Zhang T, Cheng JF, Kim Y, Grimwood J, Schmutz J, Dickson M, Noonan JP, Zhang MQ, Myers RM, Maniatis T (2001). Comparative DNA sequence analysis of mouse and human protocadherin gene clusters. Genome Res.

[B14] Yanase H, Sugino H, Yagi T (2004). Genomic sequence and organization of the family of CNR/Pcdhalpha genes in rat. Genomics.

[B15] Sugino H, Hamada S, Yasuda R, Tuji A, Matsuda Y, Fujita M, Yagi T (2000). Genomic organization of the family of CNR cadherin genes in mice and humans. Genomics.

[B16] Noonan JP, Grimwood J, Danke J, Schmutz J, Dickson M, Amemiya CT, Myers RM (2004). Coelacanth genome sequence reveals the evolutionary history of vertebrate genes. Genome Res.

[B17] Vandepoele K, De VW, Taylor JS, Meyer A, Van de PY (2004). Major events in the genome evolution of vertebrates: paranome age and size differ considerably between ray-finned fishes and land vertebrates. Proc Natl Acad Sci U S A.

[B18] Christoffels A, Koh EG, Chia JM, Brenner S, Aparicio S, Venkatesh B (2004). Fugu genome analysis provides evidence for a whole-genome duplication early during the evolution of ray-finned fishes. Mol Biol Evol.

[B19] Tada MN, Senzaki K, Tai Y, Morishita H, Tanaka YZ, Murata Y, Ishii Y, Asakawa S, Shimizu N, Sugino H, Yagi T (2004). Genomic organization and transcripts of the zebrafish Protocadherin genes. Gene.

[B20] Noonan JP, Grimwood J, Schmutz J, Dickson M, Myers RM (2004). Gene conversion and the evolution of protocadherin gene cluster diversity. Genome Res.

[B21] Venkatesh B (2003). Evolution and diversity of fish genomes. Curr Opin Genet Dev.

[B22] Aparicio S, Chapman J, Stupka E, Putnam N, Chia JM, Dehal P, Christoffels A, Rash S, Hoon S, Smit A, Gelpke MD, Roach J, Oh T, Ho IY, Wong M, Detter C, Verhoef F, Predki P, Tay A, Lucas S, Richardson P, Smith SF, Clark MS, Edwards YJ, Doggett N, Zharkikh A, Tavtigian SV, Pruss D, Barnstead M, Evans C, Baden H, Powell J, Glusman G, Rowen L, Hood L, Tan YH, Elgar G, Hawkins T, Venkatesh B, Rokhsar D, Brenner S (2002). Whole-genome shotgun assembly and analysis of the genome of Fugu rubripes. Science.

[B23] Jaillon O, Aury JM, Brunet F, Petit JL, Stange-Thomann N, Mauceli E, Bouneau L, Fischer C, Ozouf-Costaz C, Bernot A, Nicaud S, Jaffe D, Fisher S, Lutfalla G, Dossat C, Segurens B, Dasilva C, Salanoubat M, Levy M, Boudet N, Castellano S, Anthouard V, Jubin C, Castelli V, Katinka M, Vacherie B, Biemont C, Skalli Z, Cattolico L, Poulain J, De B, Cruaud C, Duprat S, Brottier P, Coutanceau JP, Gouzy J, Parra G, Lardier G, Chapple C, McKernan KJ, McEwan P, Bosak S, Kellis M, Volff JN, Guigo R, Zody MC, Mesirov J, Lindblad-Toh K, Birren B, Nusbaum C, Kahn D, Robinson-Rechavi M, Laudet V, Schachter V, Quetier F, Saurin W, Scarpelli C, Wincker P, Lander ES, Weissenbach J, Roest CH (2004). Genome duplication in the teleost fish Tetraodon nigroviridis reveals the early vertebrate proto-karyotype. Nature.

[B24] Drouin G, Prat F, Ell M, Clarke GD (1999). Detecting and characterizing gene conversions between multigene family members. Mol Biol Evol.

[B25] Taguchi Y, Koide T, Shiroishi T, Yagi T (2005). Molecular evolution of cadherin-related neuronal receptor/protocadherin(alpha) (CNR/Pcdh(alpha)) gene cluster in Mus musculus subspecies. Mol Biol Evol.

[B26] Smith NG, Eyre-Walker A (2001). Synonymous codon bias is not caused by mutation bias in G+C-rich genes in humans. Mol Biol Evol.

[B27] Galtier N, Piganeau G, Mouchiroud D, Duret L (2001). GC-content evolution in mammalian genomes: the biased gene conversion hypothesis. Genetics.

[B28] Yap AS, Brieher WM, Pruschy M, Gumbiner BM (1997). Lateral clustering of the adhesive ectodomain: a fundamental determinant of cadherin function. Curr Biol.

[B29] (2007). The National Center for Biotechnology Information. http://www.ncbi.nlm.nih.gov.

[B30] Burge C, Karlin S (1997). Prediction of complete gene structures in human genomic DNA. J Mol Biol.

[B31] (2007). UCSC genome browser. http://genome.ucsc.edu/.

[B32] Thompson JD, Gibson TJ, Plewniak F, Jeanmougin F, Higgins DG (1997). The CLUSTAL_X windows interface: flexible strategies for multiple sequence alignment aided by quality analysis tools. Nucleic Acids Res.

[B33] Perriere G, Gouy M (1996). WWW-query: an on-line retrieval system for biological sequence banks. Biochimie.

[B34] Yang Z (1997). PAML: a program package for phylogenetic analysis by maximum likelihood. Comput Appl Biosci.

[B35] Wernersson R, Pedersen AG (2003). RevTrans: Multiple alignment of coding DNA from aligned amino acid sequences. Nucleic Acids Res.

